# Reduced glucocerebrosidase activity in monocytes from patients with Parkinson’s disease

**DOI:** 10.1038/s41598-018-33921-x

**Published:** 2018-10-18

**Authors:** Farzaneh Atashrazm, Deborah Hammond, Gayathri Perera, Carol Dobson-Stone, Nicole Mueller, Russell Pickford, Woojin Scott Kim, John B. Kwok, Simon J. G. Lewis, Glenda M. Halliday, Nicolas Dzamko

**Affiliations:** 1 Brain and Mind Centre, Central Clinical School, University of Sydney, Camperdown, NSW 2050 Australia; 2 Neuroscience Research Australia, Randwick, NSW 2031 Australia; 3 Forefront Parkinson’s Disease Research Clinic, Brain and Mind Centre, University of Sydney, Camperdown, NSW 2050 Australia; 4 Bioanalytical Mass Spectrometry Facility, University of NSW, Kensington, NSW 2052 Australia; 5 School of Medical Sciences, University of NSW, Kensington, NSW 2052 Australia

## Abstract

Missense mutations in glucocerebrosidase (*GBA1*) that impair the activity of the encoded lysosomal lipid metabolism enzyme (GCase) are linked to an increased risk of Parkinson’s disease. However, reduced GCase activity is also found in brain tissue from Parkinson’s disease patients without *GBA1* mutations, implicating GCase dysfunction in the more common idiopathic form of Parkinson’s disease. GCase is very highly expressed in monocytes, and thus we measured GCase activity in blood samples from recently diagnosed Parkinson’s disease patients. Flow cytometry and immunoblotting assays were used to measure levels of GCase activity and protein in monocytes and lymphocytes from patients with Parkinson’s disease (n = 48) and matched controls (n = 44). Gene sequencing was performed to screen participants for *GBA1* missense mutations. In the Parkinson’s disease patients, GCase activity was significantly reduced in monocytes, but not lymphocytes, compared to controls, even when *GBA1* mutation carriers were excluded. Monocyte GCase activity correlated with plasma ceramide levels in the Parkinson’s disease patients. Our results add to evidence for GCase dysfunction in idiopathic Parkinson’s disease and warrant further work to determine if monocyte GCase activity associates with Parkinson’s disease progression.

## Introduction

Parkinson’s disease (PD) is a common movement disorder, with diagnostic symptoms of resting tremor, bradykinesia and muscle rigidity resulting from degeneration of dopamine producing neurons in the substantia nigra region of the midbrain. The underlying causes of PD remain unclear however, several genetic factors have been identified that associate with an increased risk of developing PD. The most common genetic risk factor for PD comprises heterozygous missense mutations in *GBA1*, the gene encoding the lysosomal enzyme beta-glucocerebrosidase (GCase). *GBA1* mutations occur in as many as 5–15% of PD cases, depending on ethnicity^1–3^, and can increase the risk of developing PD by up to 20-fold^4,5^.

The finding that *GBA1* mutations increase the risk of developing PD resulted from observation of a higher incidence of PD in patients with the lysosomal storage disorder, Gaucher’s disease, a recessive disorder also caused by *GBA1* mutations^6^. Studies of Gaucher’s disease patients have identified almost 300, mostly missense, mutations for *GBA1* that negatively impact on GCase stability and function^7^. As GCase catalyses the hydrolysis of glucosylceramide to ceramide and glucose, affected cells from Gaucher’s disease patients display lipid abnormalities^8^.

We have previously shown that GCase activity is also reduced in pathologically affected brain tissue from PD subjects without *GBA1* mutations^9^. This decreased GCase activity was not due to changes in *GBA1* mRNA expression, but was associated with impaired lysosomal function and decreased levels of ceramide. Importantly, these results suggest a broader role for GCase dysfunction in PD beyond just those carriers who have *GBA1* mutations. Indeed, in both human PD post-mortem tissue and in GCase-deficient cell and animal models, reduced GCase is associated with an increase in the pathological PD protein, α-synuclein^9–13^. Clinically, PD associated with *GBA1* mutations is also largely indistinguishable from the idiopathic form, although PD patients with *GBA1* mutations appear at greater risk of cognitive decline^14–19^, a finding consistent with a higher incidence of *GBA1* mutations in patients with dementia with Lewy bodies^20^.

There is currently much interest in the development of small molecule chaperones that can stabilise and/or increase GCase activity. Such compounds could have utility for the treatment of Gaucher’s disease, but could potentially also be therapeutic options for PD^21–23^. Consequently, a better understanding of GCase activity in PD is required. In particular, peripheral blood cells offer a convenient source of GCase for measurement and a recent study identified a significant decrease in GCase activity in whole blood samples from idiopathic PD patients^24^. However, other studies using peripheral mononuclear cells have not found a decrease in GCase activity in PD patients^25–28^. Interpretation of these outcomes is complicated due to different methodologies employed and the heterogeneous nature of the sample material. Indeed, in peripheral immune cells, GCase is most highly expressed in monocytes^29^, which comprise only a small fraction of whole blood.

To try resolve these issues, we have used the cell permeable GCase substrate 5-Pentafluorobenzoylamino Fluorescein Di-β-D-Glucopyranoside (PFB-FDglu) combined with flow cytometry and immunoblotting to determine GCase activity in specific immune cell populations isolated from PD participants relatively early in their clinical disease duration. We found that in the absence of *GBA1* missense mutations, GCase activity was significantly reduced specifically in monocytes from PD participants compared to matched controls. Further work is now required to determine the extent to which monocyte GCase activity associates with PD pathogenesis.

## Materials and Methods

### Participants

Participants were recruited with informed consent and the study was approved by both the University of Sydney, and University of New South Wales, Research Ethics Committees (Approval Numbers 2016/363 and HC16562 respectively). All methods were carried out in accordance with the relevant guidelines and regulations. Participants with PD met the Movement Disorders Society criteria for clinically established PD^30^. The control group predominantly comprised unaffected spouses of PD subjects and had no diagnosed neurological disease. Exclusion criteria for the study were a diagnosis of Gaucher’s disease, or having a first degree relative with Gaucher’s disease. For the PD group, age at diagnosis <50 years, a strong family history of PD, and a disease duration >8 years were additional exclusion criteria. Clinical severity of PD was assessed with the Hoehn and Yahr scale and the levodopa equivalent dose was calculated for PD participants on dopamine medication. Participant demographic data is provided in Table 1. Participants in this study were not fasted prior to blood collection.

**Table 1:** Demographic details for patients with Parkinson’s disease (PD) and controls.

	PD	Control
Participant number	48	44
Age (y)	67 ± 1	65 ± 1
Male % (n)	64 (31)	50 (22)
Disease duration (y)	3.5 ± 0.3	—
Disease Severity (H&Y)	1.9 ± 0.1	—
Ldopa equivalent dose	600 ± 43	—
Age at diagnosis (y)	64 ± 1	—

Values are given as mean ± standard error. Disease severity was recorded using the Hoehn and Yahr scale. Disease duration is the time since clinical diagnosis of PD.

### Isolation of peripheral blood mononuclear cells from participants

Venous blood was collected into 8 ml CPT vacutainers (BD Biosciences) and the time of blood draw recorded. Blood was then centrifuged at 1800 × g for 20 min at room temperature with plasma then collected, snap-frozen and stored at −80 °C for later analysis. The remaining peripheral blood mononuclear cell (PBMC) layer was then transferred to a new 15 ml tube and topped up to 15 ml with RPMI media supplemented with 10% heat inactivated low endotoxin fetal bovine serum (FBS) and 1 X penicillin/streptomycin solution (all from Life Technologies). PBMCs were then centrifuged at 400 × g for 7 min at room temperature, the media aspirated, and the PBMC pellet resuspended in 10 ml of supplemented RPMI media. Cell count and viability was determined using trypan blue and an automatic cell counter (Countess II-FL, Life Technologies). PBMCs were used immediately for the flow cytometry based assessment of GCase activity and immunomagnetic isolation of monocytes. 1 × 4 ml EDTA vacutainer was also filled with blood to extract buffy coat for genomic DNA extraction. For initial testing of different GCase antibodies, PBMCs were isolated from buffy coat obtained from healthy volunteer blood donors to the Red Cross blood service. Buffy coat was layered onto Ficoll Paque Plus (GE healthcare) and centrifuged at 400 × g for 30 min. The PBMC layer was then extracted and processed as above. PBMCs were used immediately to test GCase antibodies by flow cytometry and immunoblot. All studies with buffy coat were approved by the University of NSW human research ethics advisory panel (reference #HC14226).

### Flow cytometry measurement of GCase activity

GCase enzymatic activity was measured using a flow cytometry based assay, similar to assays used previously to assess GCase activity, including in samples from patients with Gaucher’s disease^29,31–34^. Briefly, 1 × 10^6^ PBMCs were treated with or without 1 mM of the GCase inhibitor Conduritol B Epoxide (CBE) (Sigma-Aldrich) for 1 hour at 37 °C. This was followed by the addition of 0.75 mM of lysosomal GCase substrate, PFB-FDglu (Life Technologies) for 30 min at 37 °C. At the end of the incubation, 1 mL of ice-cold flow buffer (1 X PBS, 1 mM EDTA, 25 mM HEPES, 1% heat inactivated FBS, pH 7.0) was added and the cells pelleted by centrifugation at 350 × g for 5 min. Cells were then resuspended in flow buffer and stained with anti-CD14 monoclonal antibody conjugated to PEcy7, anti-CD3 monoclonal antibody conjugated to V450, and anti-CD19 monoclonal antibody conjugated to APC (BD Biosciences) for 30 min at 4 °C. The appropriate isotype negative control antibodies were included for all flow cytometry studies. Cells were then washed with flow buffer and finally resuspended in 200 μl of flow buffer containing 0.5% paraformaldehyde for analysis. CD3 positive T-cells, CD14 positive monocytes and CD19 positive B-cells were gated, and fluorescein intensity measured using a FACS CantoII or Fortessa X-20 cytometer (both BD biosciences). Data were analysed using FlowJo software version 10.2 (Tree Star Inc.). GCase activity was defined as the median fluorescence intensity of cells treated without CBE, divided by the median fluorescence intensity of cells treated with CBE.

### Monocyte and lymphocyte isolation

Monocytes were isolated from PBMCs by positive selection using magnetic activated cell sorting (Miltenyi Biotech) as per the manufacturers’ protocol. In brief, 1–2 × 10^7^ PBMCs were collected and incubated with CD14 MicroBeads (Miltenyi Biotech) for 15 min at 4 °C. Cells were washed and resuspended in MACS buffer and used for magnetic sorting. The use of positive selection for monocytes also allowed us to collect the unlabelled cells representing lymphocytes, that were washed through the column and collected. The magnetically labelled monocytes were then expelled by plunger and collected. Cells were spun down and the monocyte and lymphocyte pellets were lysed with buffer consisting of 50 mM Tris.HCL pH 7.5, 1 mM EGTA, 1 mM EDTA, 1 mM sodium orthovanadate, 50 mM sodium fluoride, 5 mM sodium pyrophosphate, 0.27 M sucrose, 1 mM benzamidine, 1 mM PMSF and 1% (v/v) Triton X-100. The lysates were snap frozen and stored at −80 °C until immunoblot analysis.

### siRNA knockdown of *GBA1* for antibody validation by immunoblot and flow cytometry

Human embryonic kidney 293 (HEK293) cells were grown in Dulbecco’s modified Eagle medium (DMEM) supplemented with 10% low endotoxin foetal bovine serum and 1X penicillin/streptomycin solution (all Life Technologies) until 70% confluent. Cells were then transfected with *GBA1*-siRNA (Dharmacon, Cat. No. L-006366-00-0005) using Lipofectamine 3000 (Life Technologies) according to the manufacturers’ instructions and incubated for 48 h. After this time cells were either collected by detaching with trypsin for flow cytometry assessment of GCase protein, or lysed in cell lysis buffer for immunoblot analysis of GCase protein. For immunoblotting, the GCase protein was first deglycosylated using PNGase F (New England Biolabs) according to manufacturers’ instructions. More details on flow cytometry, and deglycosylation are available in the supplementary methods.

### Immunoblotting

Deglycosylated monocyte or lymphocyte lysates were separated with 4–12% Novex Tris-glycine gels (Life Technologies) and transferred onto nitrocellulose membrane (Biorad). Membranes were blocked with 5% skim milk powder in Tris buffered saline with 0.1% (v/v) Tween 20 (TBST). Membranes were cut into strips based on molecular weight markers and probed overnight at 4 °C for GCase (Abnova, diluted 1:1000) and β-actin (Abcam, diluted 1:10,000) as a loading control. Membranes were then washed in TBST (5 × 10 min) and incubated with anti-rabbit horseradish peroxidase (HRP) secondary antibody (Abcam) at 1:5000 dilution in 2.5% skim milk in TBST or anti-mouse Alexa Fluor 647 fluorescent secondary antibody (Abcam) in TBST for 2 h. A Chemidoc MP Imaging system (Biorad) was used for detection and immunoblot quantitation was performed using Imagelab software 5.2.1 (Biorad). The intensity of each protein band was quantified and expressed as arbitrary units standardized to β-actin. Cropped GCase and β-actin immunoblots from the control and PD participants are shown in the figures with example images of the uncropped immunoblots available as Supplementary Fig. S1.

### *GBA1* sequencing

Genomic DNA was extracted from frozen buffy coats using a QIAamp DNA blood Micro Kit (Qiagen) according to the manufacturers’ protocol. *GBA1* sequencing was performed as we have described previously^9^. More details are available in the supplementary methods.

### Plasma ELISA assays

Total α-synuclein was measured using an enzyme-linked immunosorbent assay kit (BioLegend Cat. No. 844101, previously Covance Cat. No. SIG-38974) according to the manufacturers’ protocol. Reference standards and 1:40 diluted plasma samples were added into antibody-coated wells in duplicate. Plates were read on a CLARIOstar plate reader (BMG Labtech) and concentrations of α-synuclein were interpolated from the 4-parameter regression standard curve. As red blood cell lysis can artificially elevate α-synuclein levels in plasma, plasma haemoglobin levels were also measured by sandwich ELISA (Abcam Cat. No. ab157707) according to manufacturers’ instructions. The average haemoglobin level in plasma for all samples was 49.87 ± 4.8 μg/ml.

### Mass spectrometry measurement of plasma ceramide

HPLC-grade chloroform, methanol and isopropanol (Sigma Aldrich), and glass pipettes, tubes and vials were used for lipid extraction. Internal lipid standards (Avanti Polar Lipids Inc., Alabaster, AL, USA) were added to thawed plasma samples and lipids were then extracted as described previously^35^. Lipid extracts were analyzed using a Q-Exactive Plus Mass Spectrometer coupled to a U3000 UPLC system (ThermoFisher Scientific) as described previously^35^. Chromatography was performed at 60 °C on a Waters CSH C18 UHPLC column 2.1 × 100 mm, 1.8 μM with a VanGuard guard column. Data were analyzed using LipidSearch software 4.1.16 and relative abundance of ceramide was obtained from peak areas normalized to internal standards. Fifty-nine species of ceramide were detected and these were analysed at both an individual level, and summed together to give a measure of total ceramide.

### Statistical analysis

All statistical analyses were performed using SPSS Statistics software (IBM) and statistical significance set at p < 0.05. For comparisons between two groups, either Student’s t-test or univariate analysis was used as indicated. When univariate analysis was performed, age and gender were included as covariates. Discriminant and receiver operator curve analyses were used to further compare monocyte GCase activity and ceramide levels between the control and PD participants. Spearman correlation analyses were used to determine any associations between monocyte GCase activity and demographic variables. Graphs were generated using Prism software ver. 7.0 (GraphPad).

## Results

### In house validation of flow cytometry for measuring GCase activity

**Figure 1:**
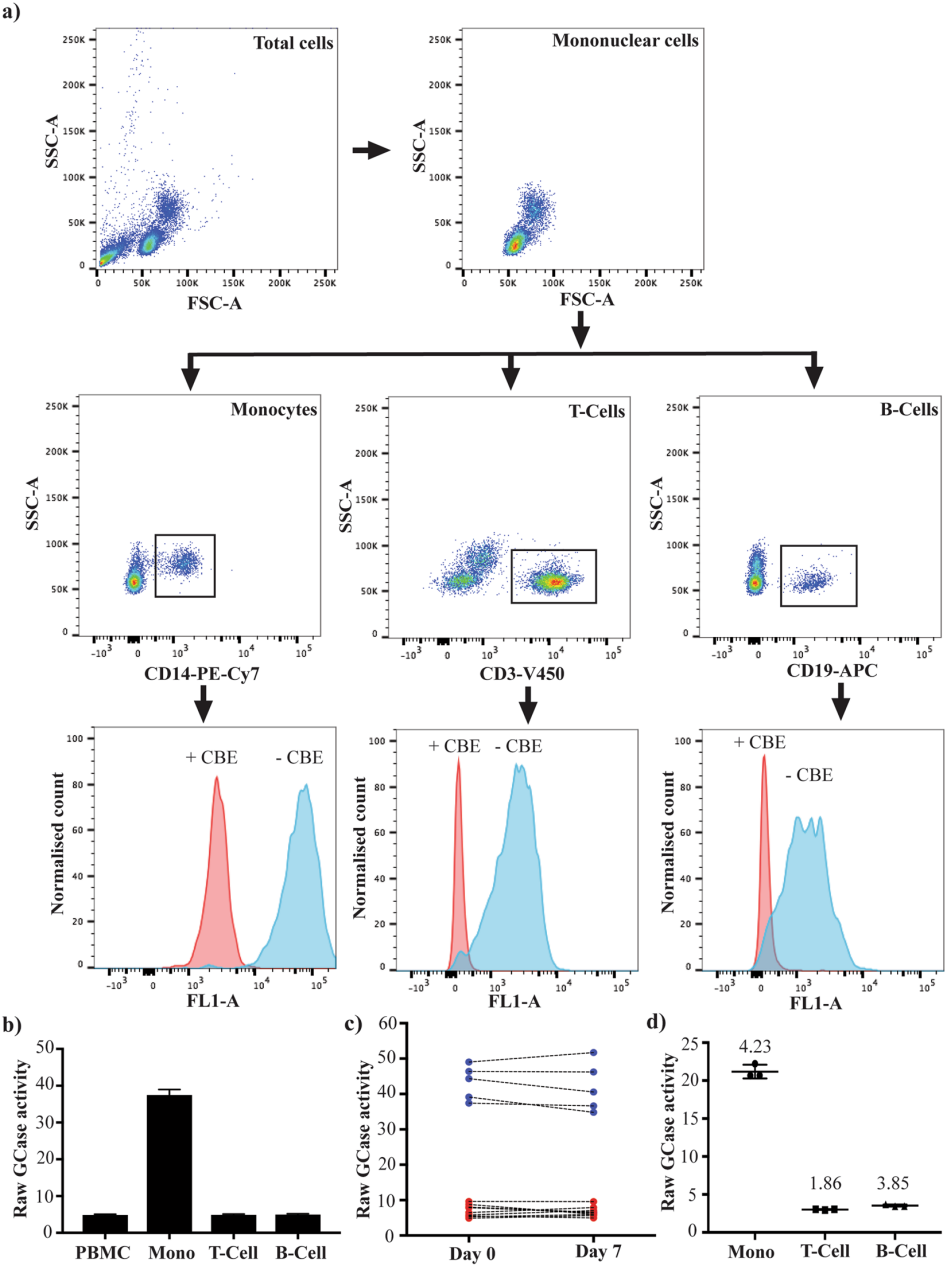
Measuring GCase activity by flow cytometry. (**a**) Peripheral blood mononuclear cells were treated with the cell-permeable lysosomal GCase substrate PFB-FDGlu, in both the presence and absence of the GCase inhibitor CBE. Flow cytometry was then used to gate on CD14- positive monocytes, CD3-positive T-cells and CD19-positive B-cells. The median fluorescence intensity due to the metabolism of PFB-FDGlu was measured in the FL1 channel for both the inhibitor and non-inhibitor treated conditions in the three different cell populations. Raw GCase activity was defined as the fluorescence intensity without CBE treatment, divided by the fluorescence intensity with CBE treatment. A representative example of the gating strategy and fluorescence results is shown. (**b**) A representative example comparing the raw GCase activity in the different immune cell populations, or when all populations are combined (PBMCs). Data are mean ± SEM, n = 3. (**c**) GCase activity was measured in monocytes (blue dots) and T and B cells (red dots) in the same 5 healthy blood donors 7 days apart. Dashed lines indicate samples from the same person. (**d**) Triplicate samples from the same blood donor were independently treated with PFB-GLU and with and without CBE and GCase activity assessed by flow cytometry. Data are mean ± SEM. The numbers represent the coefficient of variance between the technical replicate measures. Data is representative of three independent biological replicates.

Using PBMCs from healthy donors, we optimised a gating strategy to simultaneously measure the median fluorescence intensity resulting from the metabolism of PFB-FDglu, in CD14 positive monocytes, CD19 positive B-cells and CD3 positive T-cells (Fig. 1a). Positive fluorescence could be detected in the FL1 channel for all three cell populations. Importantly, incubation of the PBMCs with the GCase inhibitor CBE (Fig. 1a), or the lysosomal inhibitor bafilomycin (Supplementary Fig. S2) both ameliorated the fluorescent PFB-FDglu signal indicating specificity for lysosomal GCase. For further experiments, GCase activity was then defined as the median fluorescence intensity due to PFB-FDglu metabolism in cells without CBE, divided by the median fluorescence intensity of cells treated with CBE (i.e. the background). As expected, simultaneous comparison of the different cell populations clearly showed that the highest GCase activity was found in monocytes (Fig. 1b). We next assessed the short-term stability of GCase activity measurements in the different immune cell populations by taking blood samples from healthy donors one week apart. Over this time, the GCase activity was stable (Fig. 1c). Replicate measures from the same donors also indicated a low within sample coefficient of variance (Fig. 1d).

### Validation of antibodies for measuring GCase protein

**Figure 2:**
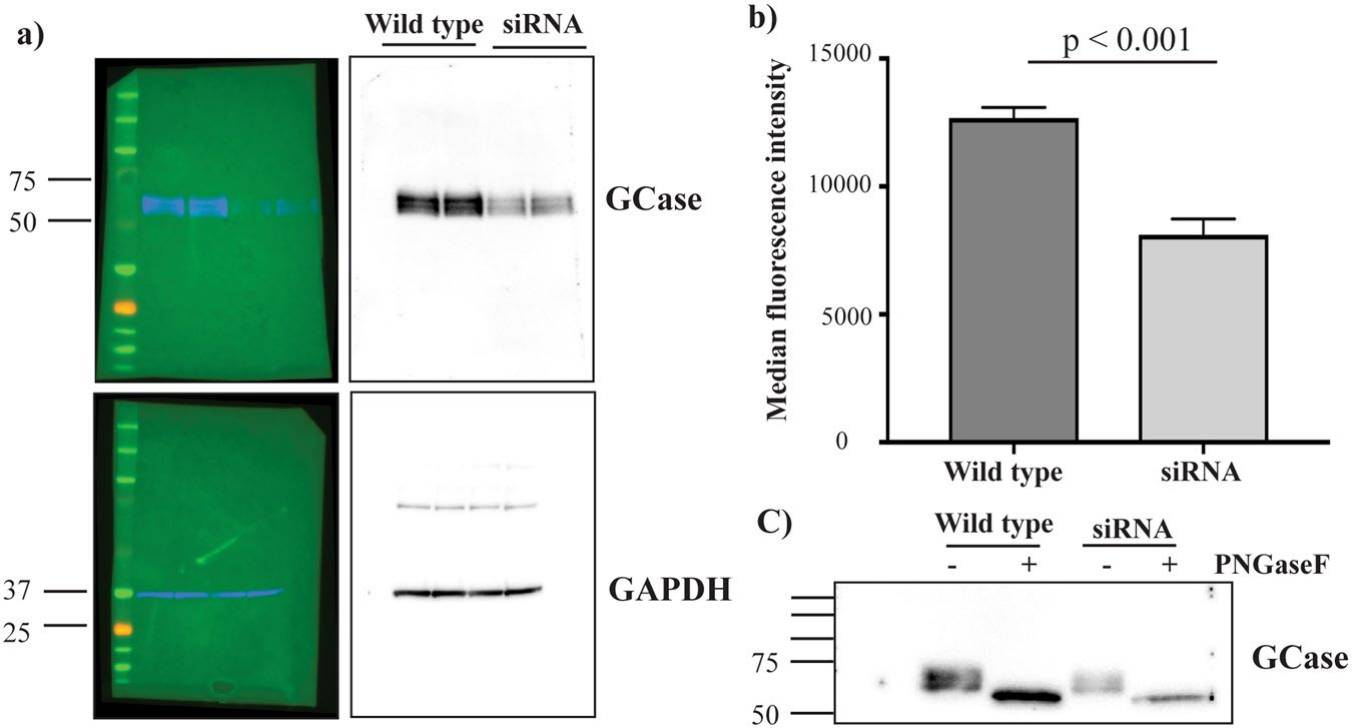
siRNA knockdown of GCase in HEK293 cells. (**a**) Representative full length immunoblot with the Abnova antibody following siRNA-mediated knockdown of GCase in HEK293 cells. Fluorescence images are included to show molecular weight markers. (**b**) Flow cytometry measurement of PFB-FDglu metabolism in HEK293 cells with and without siRNA knockdown of GCase. Data are mean ± SEM, n = 3. (**c**) Representative immunoblot using the Abnova antibody following treatment of wild type and GCase knockdown HEK293 lysates with and without PNGase F. The full-sized strip that was immunoblotted is shown.

As changes in the metabolism of PFB-FDglu by GCase may simply reflect changes in the total levels of GCase protein, we also aimed to measure total GCase protein. We tested three GCase antibodies from three different companies (Abcam: ab175869, Abnova: H00002629-M01 and Novus: MAB7410) for both flow cytometry and immunoblot. The antibody from Abnova gave the correct sized ~56 kDa band in immunoblot, but did not give a positive signal in flow cytometry under any conditions tested (Supplementary Fig. S3a). In contrast, the Abcam antibody gave a positive flow cytometry signal, but on immunoblot the band was closer to 100 kDa (Supplementary Fig. S3b). The Novus antibody did not give a robust enough signal in either immunoblot or flow cytometry under any conditions tested (Supplementary Fig. S3c). To better determine the specificity of the antibodies for GCase we used siRNA to knock down GCase protein in HEK293 cells. Immunoblotting with the Abnova antibody clearly demonstrated a loss of GCase protein in the knockdown cells (Fig. 2a), that also resulted in reduced GCase activity (Fig. 2b). The same cells were also used to simultaneously assess the specificity of the Abcam antibody, however, in both immunoblot and flow cytometry the signal was still similar in both the wild type and GCase knockdown cells, indicating non-specific binding (Supplementary Fig. S3d). Further optimisation efforts with different fix and permeabilizing conditions failed to improve these results. Consequently, we proceeded to use immunoblot to assess total levels of GCase protein using the siRNA knockdown validated Abnova antibody. To improve the quality of immunoblots for quantification, we added a deglycosylation step to remove N-linked oligosaccharides from the GCase protein. This had the desired effect of compacting the GCase band for more accurate quantification (Fig. 2c).

### Monocyte GCase activity is reduced in PD patients

**Figure 3:**
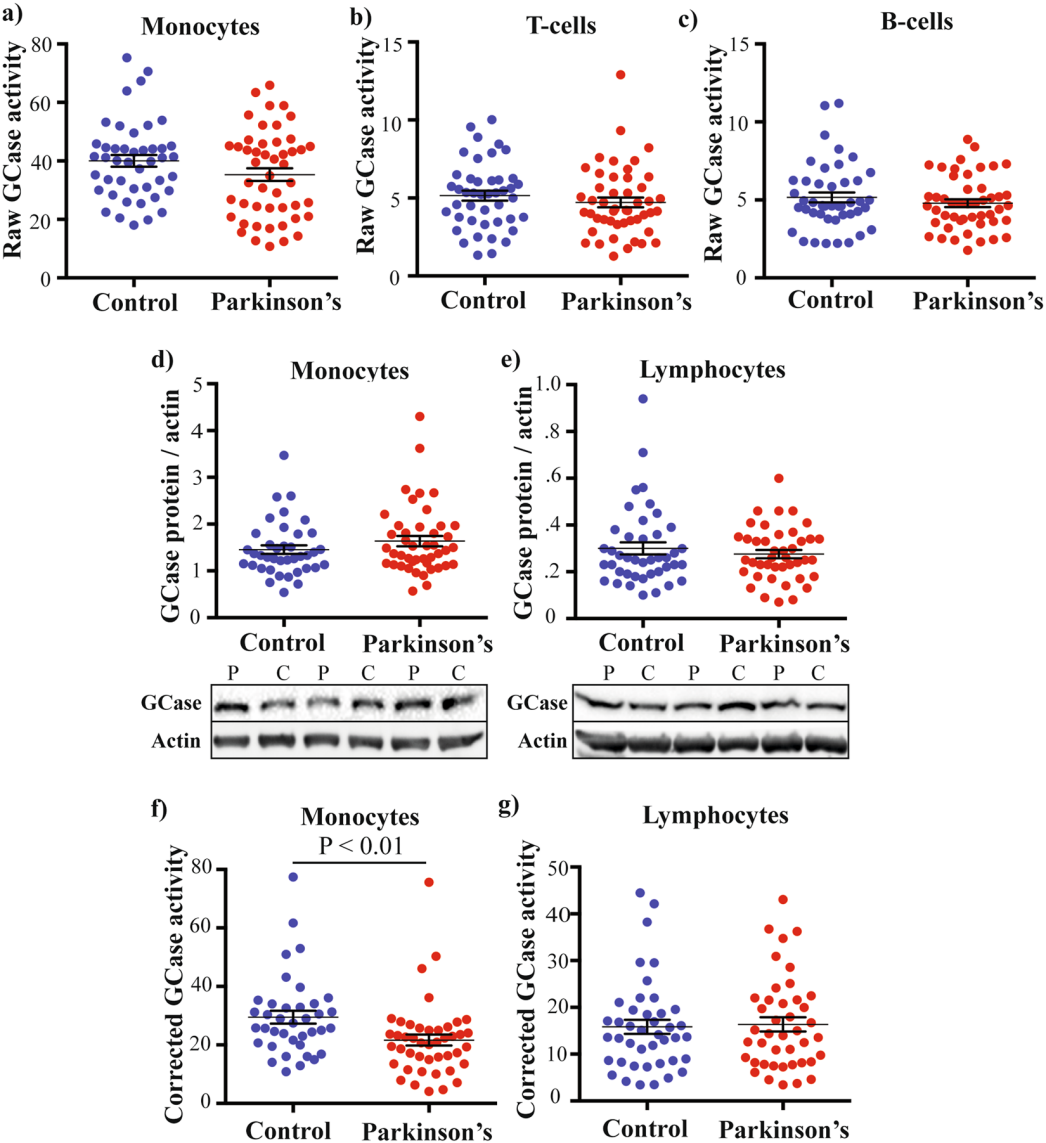
GCase activity and protein levels in mononuclear cells from control and Parkinson’s disease (PD) participants. Flow cytometry was used to measure the raw GCase activity in monocytes (**a**) T-cells (**b**) and B-cells (**c**) from controls (blue dots, n = 44) and patients with PD (red dots, n = 48). It is defined as raw GCase activity as it has not yet been corrected for GCase protein levels. At the completion of the study, monocytes (**d**) and lymphocytes (**e**) from the same controls and PD cases were immunoblotted for total GCase protein, and the levels normalised to a beta-actin loading control. Representative immunoblots are shown with the larger uncropped immunoblots available as Supplementary Fig. S1. The raw GCase activity was then divided by the GCase protein level for each participant, to give a corrected GCase activity for both monocytes (**f**) and lymphocytes (**g**). For all graphs, data are mean ± SEM with dots indicating individual data points. The control and PD groups were compared using students t-test.

We next proceeded to measure GCase activity and protein in recruited PD participants and controls. To determine if differences in peripheral GCase activity could be detected early after diagnosis, we recruited PD participants less than eight years since diagnosis. The demographic data of the cohort is shown in Table 1. All PD participants met the criteria for a clinical diagnosis of disease, and almost all were Hoehn and Yahr stage 2. As above, control and PD participant PBMCs were treated with and without CBE and the difference in median fluorescence intensity that resulted from the metabolism of PFB-FDglu by lysosomal GCase was calculated. We found no significant difference in this raw activity measure between the two groups in monocytes (Fig. 3a), B-cells (Fig. 3b) or T-cells (Fig. 3c). There was also no significant difference in GCase protein levels in monocytes (Fig. 3d) or lymphocytes (Fig. 3e) between the two groups. However, when the monocyte median fluorescence intensity was corrected for monocyte GCase protein levels for each participant, there was a significant reduction in the PD patient cells (28% decrease, p < 0.01, Fig. 3f). We then re-gated the B-cell and T-cell fluorescence data to give a combined lymphocyte group. This was also then corrected for lymphocyte levels of GCase protein, but there was still no significant difference between the two groups (Fig. 3g). These results suggest a specific decrease in GCase activity in monocytes from PD patients, and suggest that correcting the GCase activity assays for total protein levels is important. For all subsequent analyses, the GCase activity values that had been corrected for GCase protein levels were used.

### Monocyte GCase activity is reduced in *GBA1* missense mutation carriers

**Figure 4:**
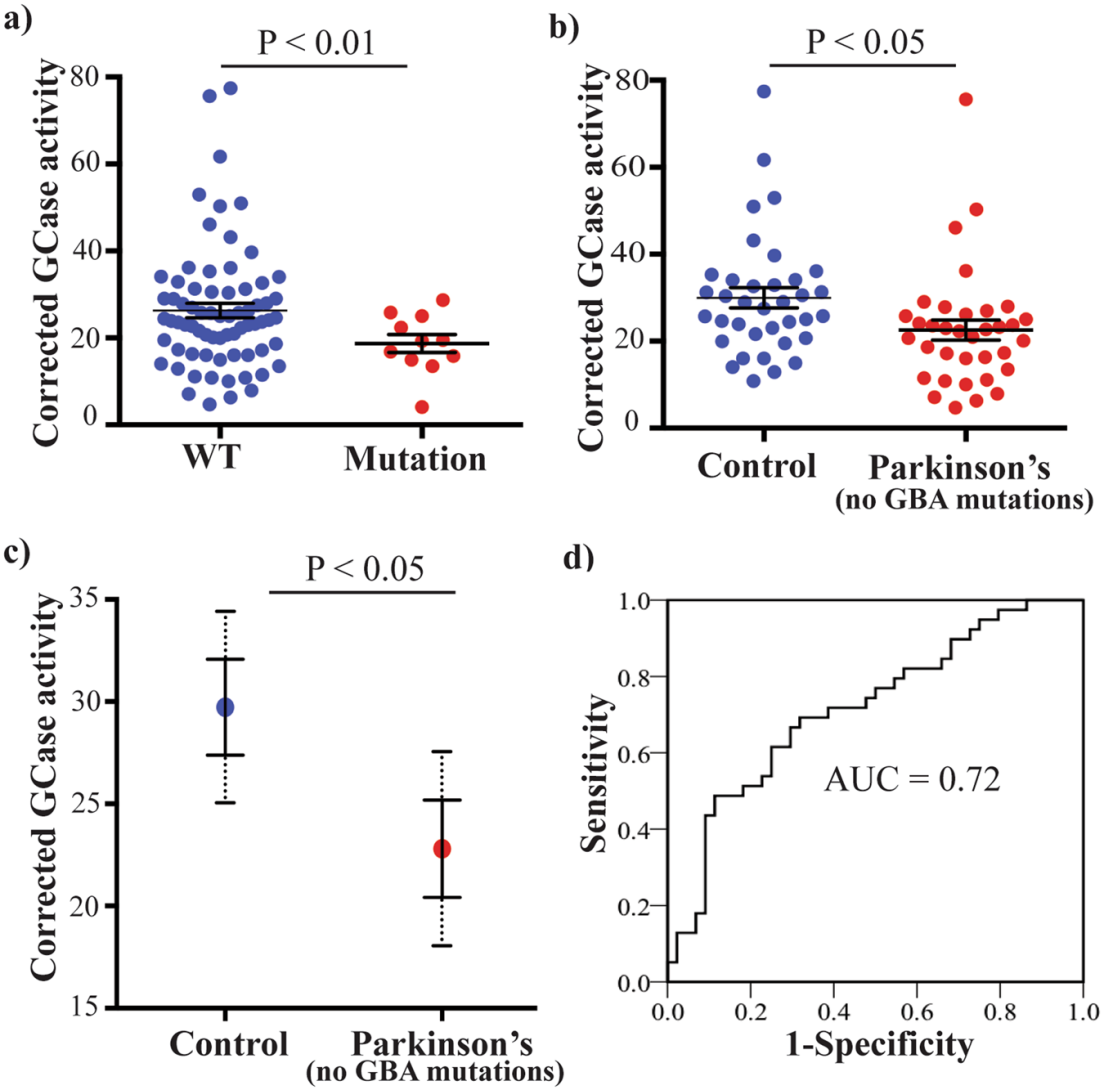
Reduced GCase activity in monocytes from patients with Parkinson’s disease (PD). (**a**) Following sequencing, participants were split into wild type (blue circles, n = 80) or *GBA1* missense mutation (red circles, n = 12) groups, and GCase activity compared by t-test. Data are mean ± SEM with dots indicating individual data points (**b**) Participants with *GBA1* mutations were then removed from the analysis and the GCase activity in controls (blue circles, n = 41) and PD patients (red circles, n = 39) was again compared by t-test. Data are mean ± SEM with dots indicating individual data points (**c**) A univariate analysis was performed to determine if monocyte GCase activity was still reduced in the PD group when age and gender were included as covariates. Data are estimated marginal means ± SEM with the 95% confidence intervals indicated by dashed lines. (**d**) Receiver operator characteristic (ROC) curve indicating the sensitivity and specificity for monocyte GCase activity to classify PD.

We then sought to determine if differences in GCase activity could be explained by the presence of *GBA1* missense mutations, many of which impair GCase activity. Sequencing of the *GBA1* gene uncovered twelve missense mutation carriers, nine in the PD group and three in the control group (Table 2). We compared the GCase activity between mutation and non-mutation carriers, and as expected, the activity was significantly reduced in the mutation carrier group (29% decrease, p < 0.01, Fig. 4a).

**Table 2:** Characteristics of the *GBA1* gene mutations identified.

Allele Name	rs Number	cDNA	Exon		PD cases (n = 48)	controls (n = 44)	Pathological significance
					Carrier % (n)	Carrier % (n)	
**All GBA1 variants**	—	—		—	**18.75% (9)**	**6.8% (3)**	—
L444P	rs421016	c.1448 T > C	HT	10	2.1% (1)	—	**Pathogenic:** Gaucher’s disease Parkinson disease Dementia, Lewy body
E326K	rs2230288	c.1093 G > A	HT	8	2.1% (1)	6.8% (3)	**Conflicting;** Gaucher’s disease Parkinsonism
T369M	rs75548401	c.1223 C > T	HT	8	8.4% (4)	—	**Conflicting;** Parkinson disease, late-onset
D380N	—	c.1255 G > A	HT	9	2.1% (1)	—	Gaucher’s Disease
G82R	—	c.361 G > A	HT	4	2.1% (1)	—	Unknown
W184R^*^ N188K^*^ V191G^*^ S196P^*^ G202R^*^ F213I^*^ Truncated GBA	rs61748906 rs61748906 rs381427 rs1064644 rs398123534 rs381737 —	c.667 T > C c.681 T > C c.689 T > G c.703 T > C c.721 G > A c.754 T > A —	HM HM HM HM HM HM	6 6 6 6 6 6 Intron 6: 321 bp del (Possible gene-pseudogene recombinant)	2.1% (1)	—	**Pathogenic**, Gaucher’s disease **Pathogenic**, Gaucher’s disease Gaucher’s disease **Pathogenic**, Gaucher’s disease **Pathogenic**, Gaucher’s disease **Pathogenic**, Gaucher’s disease Unknown

Sequencing of *GBA1* was performed to identify missense mutations that may be pathogenic for Parkinson’s disease. Pathological significance indicators were taken from the ClinVar database. Allele names follow the common nomenclature and apply to the processed protein, not including the 39-residue signal peptide. cDNA sequence numbering starts with the adenine of the first translated ATG start codon (GenBank reference sequence NM_000157.3). Alterations indicated with a * were all present in the same participant and correspond to sequence present in the *GBA1* pseudogene, *GBAP1*. HM = Homozygous, HT = Heterozygous for the indicated mutation. References for this table are located in the supplementary references section.

### Monocyte GCase activity is reduced in PD patients without *GBA1* missense mutations

We next removed the twelve mutation cases from the dataset and repeated the analysis of monocyte GCase activity. Importantly, a significant reduction in monocyte GCase activity was still observed in the PD participants without a *GBA1* missense mutation (25% decrease, p < 0.05, Fig. 4b). We noted that the removal of the mutation cases resulted in a significant age difference between the control and PD groups (64 ± 1 and 69 ± 1 years respectively, p < 0.05), as the *GBA1* mutation carriers with PD had an earlier age-at-diagnosis than those without (58 ± 3 and 65 ± 1 years respectively, p < 0.05). To determine if differences in age and/or gender could explain the reduced monocyte GCase activity in PD patients, we performed a univariate analysis covarying for these two variables. Again, monocyte GCase activity was still significantly reduced in the PD patients without *GBA1* mutations (24% decrease, p < 0.05, Fig. 4c) and both age and gender had no significant effect (p = 0.882 and p = 0.376 respectively). Monocyte GCase activity could also differentiate between control and PD patients using linear discriminant analysis (Wilks’ Lambda = 0.930, Chi-square = 4.941, p < 0.05), with receiver operator characteristic analysis showing a limited ability of monocyte GCase activity to classify PD (AUC = 0.72, p < 0.001, Fig. 4d).

### Monocyte GCase activity did not associate with available clinical variables or time of blood collection

We next performed correlation analyses to determine if monocyte GCase activity associated with available clinical variables in the PD cohort. However, there was no significant correlation between monocyte GCase activity and age at diagnosis, disease duration, disease severity or dopamine medication (Supplementary Table S4). To initially assess the effect of time of day of blood collection on monocyte GCase activity, we set 8:00 am as a zero time point and calculated the elapsed minutes since 8:00 am until blood collection for each participant. When calculated in this manner, the average time for blood collection was 181 ± 12 min from 8am for controls (ie, 11:01 am), and 181 ± 14 min from 8am for the Parkinson’s participants (ie. 11:00 am) (p = 0.983 by t-test between the two groups). There was also no significant correlation between the elapsed time from 8:00 am until blood collection and monocyte GCase activity (Supplementary Table S4).

### Monocyte GCase activity correlates with plasma ceramide levels

**Figure 5:**
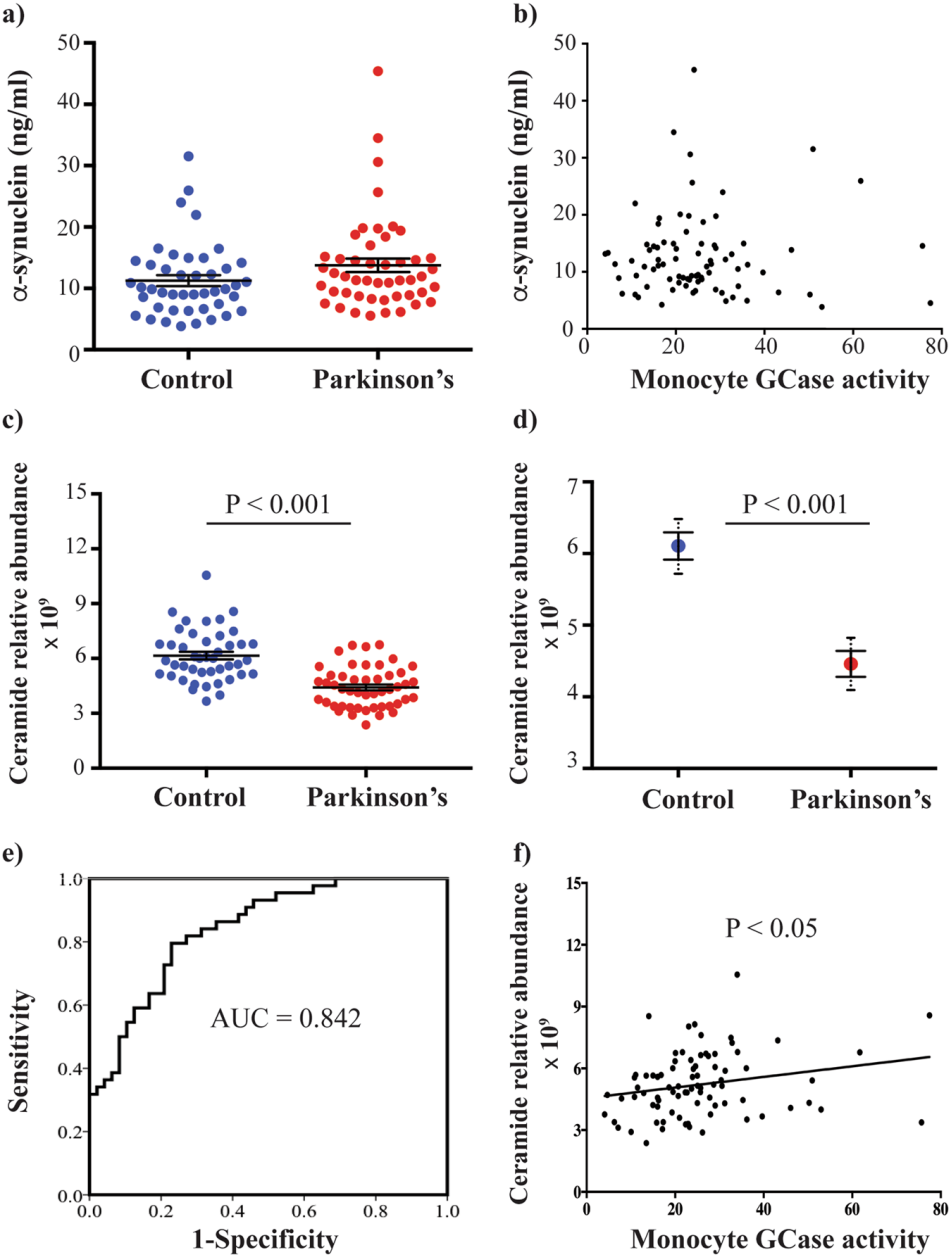
Reduced GCase activity in monocytes from patients with Parkinson’s disease (PD). (**a**) Levels of plasma monomeric α-synuclein in controls (blue circles, n = 44) and PD patients (red circles, n = 48). data are mean ± SEM with dots indicating individual data points (**b**) Scatterplot showing the relationship between plasma α-synuclein and monocyte GCase activity. (**c**) Levels of total plasma ceramide in controls (blue circles, n = 44) and patients with Parkinson’s disease (red circles, n = 48). data are mean ± SEM with dots indicating individual data points (**d**) A univariate analysis was performed to determine if total plasma ceramide was still reduced in the PD group when age and gender were included as covariates. Data are estimated marginal means ± SEM with the 95% confidence intervals indicated by dashed lines. (**e**) Receiver operator characteristic (ROC) curve indicating the sensitivity and specificity for total plasma ceramide to classify PD. (**f**) Correlation between total plasma ceramide and monocyte GCase activity.

As we previously observed reduced GCase activity associated with elevated α-synuclein and reduced ceramide, we also measured plasma levels of these in the control and PD patients. The plasma levels of monomeric α-synuclein were not significantly different between the two groups (Fig. 5a) and plasma α-synuclein did not correlate with monocyte GCase activity (rho = 0.233, p = 0.128, Fig. 5b). Mass spectrometry analysis of ceramides identified 59 species, 36 of which were significantly reduced in the PD patient plasma compared to controls (Supplementary Table S5). For each participant, the individual quantified ceramide species were summed together to give a total plasma ceramide level, which was significantly decreased in the PD group compared to controls (28% decrease, p < 0.001, Fig. 5c). The decreased ceramide in PD patients remained significant after covarying for age and gender (27% decrease, p < 0.001, Fig. 5d). In the univariate analysis, gender had a significant effect on ceramide levels (p < 0.05), indicating that controlling for gender is important for interpreting ceramide results. Total plasma ceramide levels could also differentiate between control and PD patients using linear discriminant analysis (Wilks’ Lambda = 0.930, Chi-square = 4.941, p < 0.05), with receiver operator characteristic analysis showing a better ability of plasma ceramide to classify PD (AUC = 0.84, p < 0.001, Fig. 5e). Finally, plasma levels of total ceramide were significantly correlated with GCase activity (rho = 0.259, p < 0.05, Fig. 5f).

## Discussion

Homozygous and compound heterozygous *GBA1* missense mutations that impair GCase activity cause Gaucher’s disease^36^. Gaucher’s patients also exhibit a higher incidence of PD^37^, and subsequent genetic analyses of PD patients have demonstrated that *GBA1* missense mutations constitute a relatively common risk factor for developing PD^4^. Importantly, impaired GCase activity is also found in affected brain tissue from PD patients that do not have *GBA1* mutations^9^, suggesting that impaired GCase activity may play a role in the more common idiopathic form of PD. To further examine GCase activity in PD, we have used flow cytometry to measure GCase activity in peripheral immune cells. We found a significant decrease in GCase activity in PD participant monocytes, but not in lymphocytes. Importantly, the decrease in monocyte GCase activity was observed in PD participants that did not have *GBA1* missense mutations.

The selective decrease in monocytes may be important as other recent studies measuring GCase activity in PD participants have predominantly used total leukocyte preparations. Monocytes only comprise a few percent of total leukocytes and thus it may be difficult to detect reduced GCase activity in heterogeneous cell populations. In support of this concept, Alcalay *et al*. reported a significant 6% decrease in GCase activity in total leukocytes with a sample size of 517 PD participants^24^, whilst studies with smaller sample sizes have not found a decrease in GCase activity in idiopathic PD participants^25–28^. When we combined the GCase activity of monocytes and lymphocytes together to form a peripheral blood mononuclear cell group, of which monocytes comprise ~15% of total cells, there was no longer a significant difference in GCase activity between control and PD, consistent with other studies using a similar sample size. Therefore, monocytes express the highest levels of GCase, but only comprise a small subset of peripheral blood cells, and thus studying heterogeneous cell populations may mask monocyte-specific differences in PD.

Reduced monocyte GCase activity correlated with reduced levels of total plasma ceramide, which is produced, amongst other means, by the metabolism of glucosylceramide by GCase. As a result, total plasma ceramide was also significantly reduced in the PD participants, to the same extent as the monocyte GCase activity. It is interesting to note that 36 out of 59 measured ceramide species, including the most abundant, were significantly reduced in the PD patient serum samples. These ceramide species in particular, could potentially be utilised to develop a more sensitive readout of peripheral GCase activity. However, further work is necessary to determine the exact chemistry of the ceramide species. In particular, a previous study has reported an increase in 5 out of 9 plasma ceramide species identified in PD participants compared to controls^38^. Major differences between the two studies are that our control cohort had a sample size of 44, compared to a control cohort size of 5, and that we have employed an advanced lipidomic strategy^39^, allowing the identification of 59 ceramide species compared to 9. More recently, plasma ceramide levels have also been reported as increased in PD patients carrying *GBA1* mutations compared to PD patients without *GBA1* mutations^40^. A comparison of plasma ceramide levels to controls was not performed in that study and we note that composition of the *GBA1* mutation carrier group is substantially different to the mutation carriers we identified in the present study. Over 300 mutations of varying pathogenicity have been reported for *GBA1* and the extent to which individual mutations perturb either GCase activity or lipid metabolism still requires a greater understanding. Thus, although it is plausible that a loss of GCase activity will result in a loss of its product, further work is still required to better understand the observed relationship between GCase activity and plasma ceramide levels.

One potential outcome of our study is that monocyte GCase activity could be used to stratify idiopathic PD patients into clinical trials targeting the GCase enzyme. Indeed, the first trials have been announced and are currently recruiting *GBA1* mutation carriers to assess the efficacy of the small molecule GCase-stabilizing chaperone ambroxol, and GZ/SAR402671, a small molecule that reduces availability of the GCase substrate glucosylceramide^22^. However, more work is required to determine if reduced monocyte GCase activity reflects early GCase dysfunction in the brain, and/or an association with PD progression. It should be noted that our PD cohort comprised of patients with less than 8 years disease duration, and almost all patients scored two on the Hoehn and Yahr disease severity scale. Thus, it would be important to both replicate and expand our study to encompass a more diverse range of clinical symptomology. Indeed, a number of idiopathic PD participants had monocyte GCase activity levels similar to *GBA1* mutation carriers, which are known to have increased risk of cognitive decline^15–18^. To determine if monocyte GCase activity can predict future clinical outcomes, such as progression of motor symptoms or cognitive decline, will require more comprehensive longitudinal assessment of idiopathic PD participants with high and low levels of monocyte GCase activity. It would also be of interest to assess GCase activity in prodromal PD cohorts, such as patients with idiopathic REM sleep behavior disorder that comprise a very high PD risk group^41^. As well as a replication and longitudinal assessment, we would also recommend further modifications to the GCase assay. In particular, replacing semi-quantitative immunoblotting with a more quantitative assay for measuring GCase protein would better facilitate larger scale longitudinal studies.

Finally, more work is also required to provide mechanistic insight into how monocyte GCase activity is reduced in idiopathic PD. In idiopathic PD post-mortem brain, reduced GCase activity was associated with reduced levels of the lysosomal membrane protein LAMP2^9,42^, and lysosomal dysfunction in PD brain is well documented^43^. Recent studies have suggested altered monocyte function in PD patient immune cells, particularly in regard to innate immunity^44–46^, however, it would be of interest to directly determine if underlying lysosomal dysfunction is present in PD monocytes and the extent that this associates with reduced GCase activity. This may also be important for understanding the intrinsic variation in monocyte GCase activity seen in both the control and PD groups. Whether this variation associates with inflammation or lysosomal function, or may reflect different ethnicities or diets, or is associated with non-coding *GBA1* polymorphisms or other remains to be determined.

In summary, we provide initial evidence that monocyte GCase activity is reduced in idiopathic PD participants and further replicative and longitudinal studies are required to determine if reduced monocyte GCase activity can predict future clinical outcomes and/or be used to stratify idiopathic PD patients into appropriate clinical trials.

## Electronic supplementary material

Supplementary Material

## Data Availability

The datasets generated during and/or analyzed during the current study are available from the corresponding author on reasonable request.
