# Supplementary material for: Reduced glucocerebrosidase activity in monocytes from patients with Parkinson’s disease
Source: Sci Rep. 2018 Oct 18;8:15446. doi: 10.1038/s41598-018-33921-x (PMC6193988; doi:10.1038/s41598-018-33921-x)
Supplement: Supplementary file 1 — Supplementary Material [file 41598_2018_33921_MOESM1_ESM.pdf]

Reduced glucocerebrosidase activity in monocytes from patients with Parkinson's disease.

Farzaneh Atashrazm[1,2], Deborah Hammond[1,3], Gayathri Perera[1,2], Carol Dobson-Stone[1,5], Nicole Mueller[1,2], Russell Pickford[4], Woojin Scott Kim[1,2,5], John B Kwok[1,5], Simon JG Lewis[3], Glenda M Halliday[1,2,5*], Nicolas Dzamko[1,2,5*]

1. Brain and Mind Centre, Central Clinical School, University of Sydney, Camperdown, NSW, 2050, Australia
2. Neuroscience Research Australia, Randwick, NSW, 2031, Australia
3. Forefront Parkinson's Disease Research Clinic, Brain and Mind Centre, University of Sydney, Camperdown, NSW, 2050, Australia
4. Bioanalytical Mass Spectrometry Facility, University of NSW, Kensington, NSW, 2052, Australia
5. School of Medical Sciences, University of NSW, Kensington, NSW, 2052, Australia

***Correspondence to**: Nicolas Dzamko nicolas.dzamko@sydney.edu.au or Glenda Halliday glenda.halliday@sydney.edu.au, Brain and Mind Centre Central Clinical School, University of Sydney, Camperdown, NSW, 2050, Australia

SUPPLEMENTARY METHODS

Flow cytometry measurement of GCase protein levels

Indirect flow cytometry was used to measure the protein levels of GCase in PBMCs. In brief, 2×10^6 cells were incubated with PhosFlow Fix Buffer I (BD Biosciences) for 10 min. Cells were then washed with flow buffer and incubated with FCR blocking reagent (Miltenyi Biotec) for 10 min at 4 °C. Following blocking, cells were permeabilized for intracellular staining using two different methods. In method one, BD PhosFlow™ Perm Buffer I (BD Biosciences) was added into cell suspension. In method two, cells were permeabilized with 100 µl of methanol on ice for 30 min. Cells were stained with unconjugated primary antibody (Abcam, Abnova, or Novus) for 30 min and then washed and incubated with Alexa Fluor 488 labelled secondary antibody (Abcam Cat. No. ab150073). GCase protein expression was then analysed using a FACS CantoII cytometer (BD biosciences), and flow cytometry data were analysed using FlowJo software version 10.2 (Tree Star Inc.).

De-glycosylation of GCase protein for immunoblot quantification

Cell lysates were thawed on ice and centrifuged at 12000 × g for 20 min at 4 °C and the protein concentration of the supernatant was determined by bicinchoninic assay (Thermofisher). 20 µg of monocyte protein or 100 µg of lymphocyte protein was then denatured by boiling for 10 min and de-glycosylated by incubation with 800 U PNGase F (New England Biolabs) for 2 h at 37 °C. LDS sample buffer (Thermofisher) was added to the cell lysates to stop the reaction and facilitate immunoblotting.

GBA1 sequencing

GBA1 gene exons and most intronic sequences were PCR amplified using Platinum Taq DNA polymerase (ThermoFisher) in three fragments of 1.7-3 kb in length using primers designed by

reference to the genomic sequence (GenBank J03059.1) to selectively amplify the gene and not the homologous pseudogene (GenBank J03060.1). Successful PCR was confirmed by visualising PCR products using agarose gel electrophoresis. Exosap-IT clean-up reagent (ThermoFisher) was used on the PCR products according to the manufacturers' protocol. Cleaned PCR products were sequenced with internal primers, adjacent to coding exons and exon-intron boundaries, using BigDye Terminator v3.1 Cycle Sequencing Kit (ThermoFisher). Sequencing product was purified using Ethanol/EDTA precipitation, and electrophoresed on an AB3730 DNA analyser. Data were analysed with Lasergene Seqman Pro ver. 12.2 (DNASTAR).

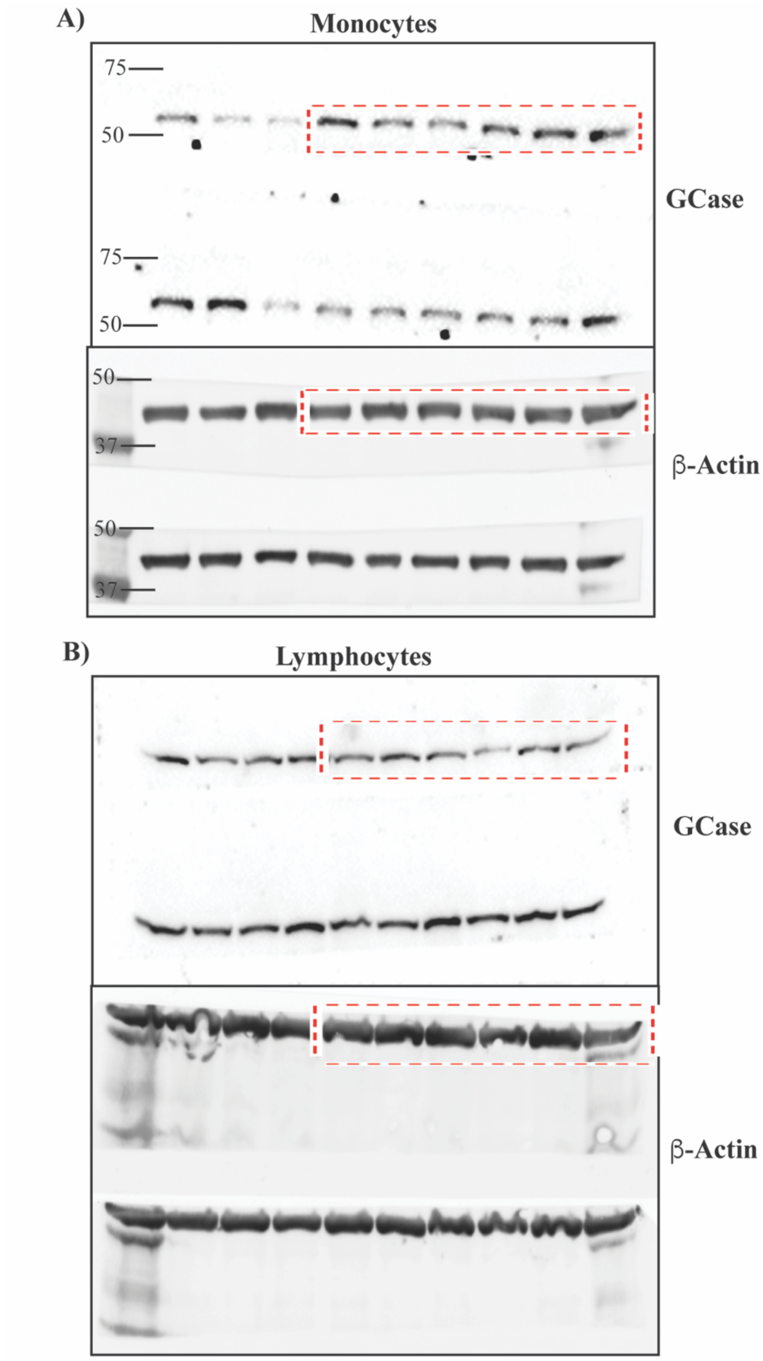

Supplementary Figure S1. Representative GCase immunoblots from participant mononuclear cells. Immunoblotting for GCase protein was performed at the end of recruitment for both monocytes **(A)** and lymphocyte **(B)** fractions. Immunoblots were cut into strips and all strips for each protein were identically processed and imaged at the same time. Representative immunoblots are shown. Dashed lines indicate cropped immunoblots presented in Figure 3.

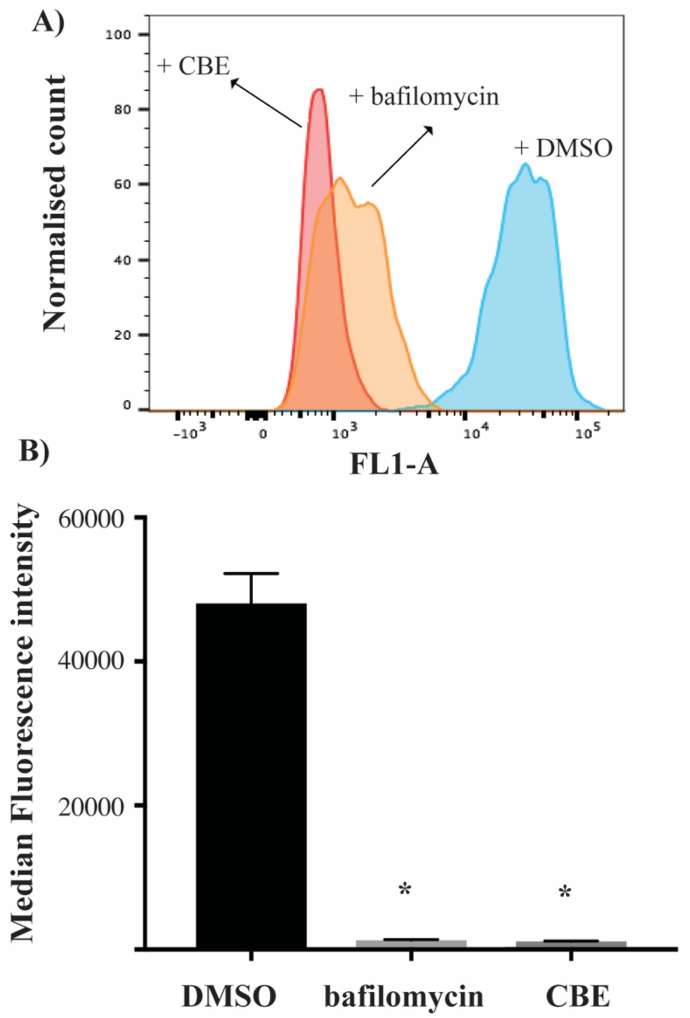

Supplementary Figure S2. Inhibition of PFB-FDglu metabolism with bafilomycin. To confirm specificity for lysosomal GCase, monocytes from healthy donors were treated with or without 1mM of selective GCase inhibitor conduritol B epoxide (CBE), or 0.2 µM of the lysosomal inhibitor bafilomycin for 1 h prior to addition of PFB-FDglu. **A)** a representative flow cytometry histogram showing that both compounds reduce the fluorescence intensity derived from PFB-FDglu metabolism. **B)** Quantification of the median florescence intensity from monocytes treated with or without CBE or bafilomycin. Data are mean ± SEM, n=3. * indicates $p < 0.05$ compared to the untreated group.

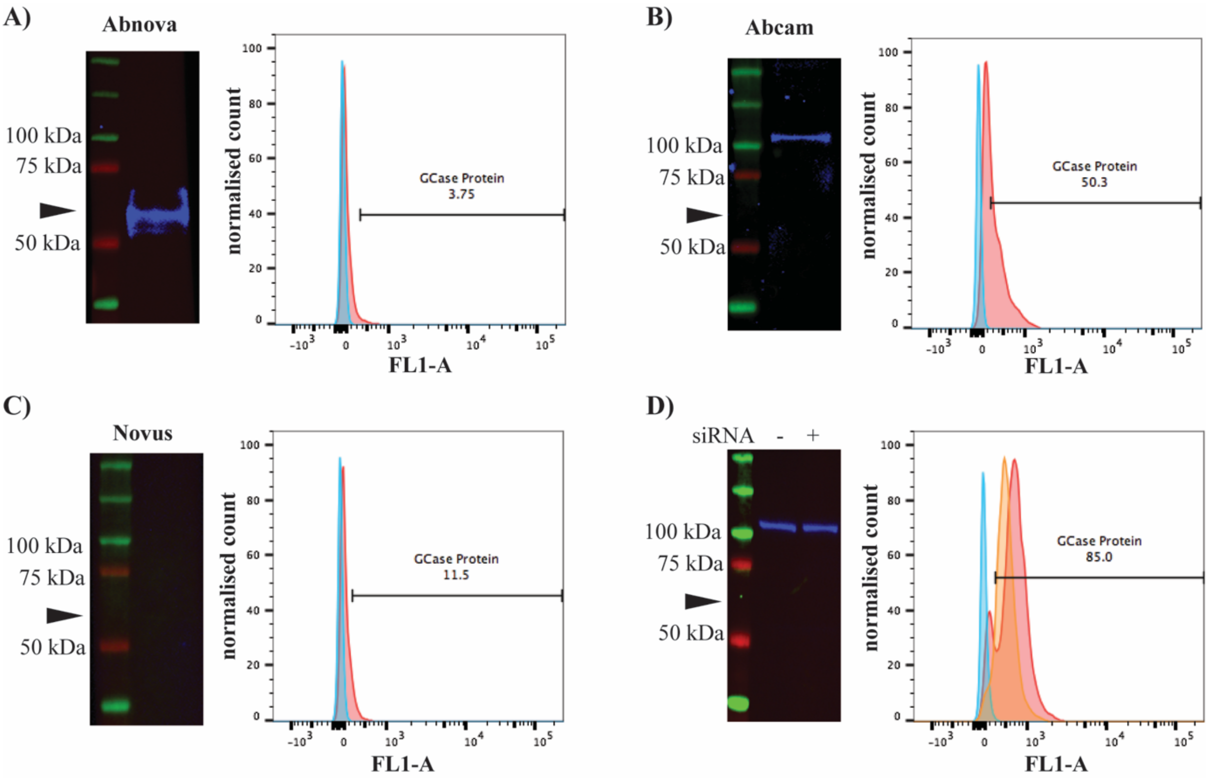

Supplementary Figure S3. Measuring GCase protein by flow cytometry. Antibodies from Abnova **(A)**, Abcam **(B)** and Novus **(C)** were used to compare the detection of GCase protein in PBMCs using immunoblotting and flow cytometry. The expected molecular weight of GCase is 56 kDa and is indicated in immunoblots with an arrow head. For flow cytometry, alexa fluor 488 (AF488) secondary antibodies were used and fluorescence measured in the FL1 channel. Red indicate primary antibody followed by AF488, blue indicates the AF488 antibody alone. Flow cytometry plots are indicative of results obtained under varying conditions of fixing and permeabilising. **D)** Flow cytometry measurement of GCase protein with the Abnova antibody in wild type (red) and siRNA knockdown HEK293 cells (orange) compared to secondary alone (blue).

		Monocyte GCase activity
**Age**	Spearman's rho	0.106
	Significance (p-value)	0.493
**Disease duration**	Spearman's rho	0.086
	Significance (p-value)	0.577
**Disease Severity**	Spearman's rho	-0.035
	Significance (p-value)	0.820
**Age at diagnosis**	Spearman's rho	0.081
	Significance (p-value)	0.601
**Dopamine medication**	Spearman's rho	0.112
	Significance (p-value)	0.470
**Time to blood draw**	Spearman's rho	0.065
	Significance (p-value)	0.615

Supplementary Table S4. Correlation between GCase activity and clinical variables in the PD cohort. Spearman correlations were performed to identify any associations between monocyte GCase activity and available clinical variables in patients with PD (n=48). Disease severity was measured using the Hoehn and Yahr scale. Dopamine medication refers to the levodopa equivalent dose. Time to blood draw was determined by the number of elapsed minutes from 8:00 am.

LipidIon	CalcMz	IonFormula	Controls	PD	% Decrease	t-test
**Cer(d18:1_12:0)**	482.46	C30 H60 O3 N1	23,632.5 ± 604.1	17,818.3 ± 580.0	24.60	**5.95E-10**
**Cer(d16:1_16:0)**	510.49	C32 H64 O3 N1	172.6 ± 18.4	165.9 ± 16.1	3.86	7.86E-01
**Cer(d32:1)**	510.49	C32 H64 O3 N1	114.4 ± 13.9	51.8 ± 6.1	54.76	**1.18E-04**
**Cer(d18:0_16:0)**	540.54	C34 H70 O3 N1	830.9 ± 38.9	746.8 ± 36.1	10.12	1.17E-01
**Cer(d18:1_16:0)**	538.52	C34 H68 O3 N1	5,078.2 ± 103.4	5,027.4 ± 134.4	1.00	7.65E-01
**Cer(d34:1)**	538.52	C34 H68 O3 N1	24,594.5 ± 1,120.4	4,292.8 ± 372.6	82.55	**3.29E-23**
**Cer(d17:1_18:0)**	552.54	C35 H70 O3 N1	573.4 ± 30.7	530.2 ± 26.1	7.53	2.87E-01
**Cer(d35:1)**	552.54	C35 H70 O3 N1	991.2 ± 49.7	126.4 ± 14.2	87.24	**3.06E-22**
**Cer(d18:0_18:0)**	568.57	C36 H74 O3 N1	593.0 ± 42.1	491.1 ± 34.6	17.18	6.50E-02
**Cer(d18:1_18:0)**	566.55	C36 H72 O3 N1	5,867.2 ± 348.4	5,274.9 ± 269.6	10.09	1.82E-01
**Cer(d18:1_18:0)**	566.55	C36 H72 O3 N1	39,373.5 ± 1,790.9	6,636.7 ± 601.1	83.14	**2.12E-23**
**Cer(d18:2_18:0)**	564.54	C36 H70 O3 N1	791.9 ± 39.5	757.0 ± 35.3	4.40	5.13E-01
**Cer(d37:1)**	580.57	C37 H74 O3 N1	1,568.1 ± 77.8	218.9 ± 24.5	86.04	**2.63E-22**
**Cer(d18:0_20:0)**	596.60	C38 H78 O3 N1	848.2 ± 55.5	674.4 ± 48.1	20.48	**2.02E-02**
**Cer(d18:1_20:0)**	594.58	C38 H76 O3 N1	8,920.9 ± 434.5	8,461.0 ± 343.8	5.16	4.09E-01
**Cer(d38:1)**	594.58	C38 H76 O3 N1	599.6 ± 182.4	696.8 ± 192.3	-16.22	7.15E-01
**Cer(d38:1)**	594.58	C38 H76 O3 N1	19,546.2 ± 869.3	3,054.8 ± 296.2	84.37	**3.63E-24**
**Cer(d18:2_20:0)**	592.57	C38 H74 O3 N1	1,044.1 ± 52.7	965.5 ± 54.3	7.53	3.02E-01
**Cer(d16:1_23:0)**	608.60	C39 H78 O3 N1	5,435.3 ± 274.6	4,724.9 ± 235.3	13.07	5.27E-02
**Cer(d39:2)**	606.58	C39 H76 O3 N1	374.3 ± 32.2	82.5 ± 10.0	77.96	**1.45E-11**
**Cer(d18:0_22:0)**	624.63	C40 H82 O3 N1	5,036.8 ± 312.5	3,717.7 ± 281.1	26.19	**2.31E-03**
**Cer(d18:0_22:0)**	624.63	C40 H82 O3 N1	778.7 ± 38.1	560.6 ± 27.6	28.02	**1.37E-05**
**Cer(d18:1_22:0)**	622.61	C40 H80 O3 N1	261.5 ± 12.5	216.6 ± 9.4	17.19	**5.25E-03**
**Cer(d16:0_24:1)**	622.61	C40 H80 O3 N1	548.3 ± 40.9	433.5 ± 42.1	20.94	5.35E-02
**Cer(d18:1_22:0)**	622.61	C40 H80 O3 N1	51,995.9 ± 2,255.7	43,614.7 ± 1,798.4	16.12	**4.69E-03**
**Cer(d20:1_20:0)**	622.61	C40 H80 O3 N1	511.6 ± 23.7	65.6 ± 9.8	87.18	**9.49E-25**
**Cer(d18:1_22:0+O)**	638.61	C40 H80 O4 N1	572.2 ± 52.6	422.4 ± 36.2	26.18	**2.14E-02**
**Cer(d18:2_22:0)**	620.60	C40 H78 O3 N1	3,392.4 ± 196.1	3,064.5 ± 168.8	9.67	2.08E-01
**Cer(d18:0_23:0)**	638.64	C41 H84 O3 N1	3,600.1 ± 218.7	2,584.7 ± 199.0	28.21	**9.09E-04**
**Cer(d18:1_23:0)**	636.63	C41 H82 O3 N1	48,261.1 ± 2,157.7	38,640.7 ± 1,654.5	19.93	**6.65E-04**
**Cer(d18:1_23:0+O)**	652.62	C41 H82 O4 N1	977.6 ± 78.7	710.0 ± 58.5	27.37	**8.01E-03**
**Cer(d18:2_23:0)**	634.61	C41 H80 O3 N1	6,549.1 ± 320.8	5,773.5 ± 287.9	11.84	7.54E-02
**Cer(d18:0_24:0)**	652.66	C42 H86 O3 N1	990.7 ± 57.7	761.4 ± 42.1	23.15	**1.92E-03**
**Cer(d18:0_24:0)**	652.66	C42 H86 O3 N1	8,012.0 ± 493.4	5,818.8 ± 469.1	27.37	**1.78E-03**
**Cer(d18:0_24:0)**	652.66	C42 H86 O3 N1	1,265.0 ± 61.8	898.2 ± 47.7	29.00	**1.04E-05**
**Cer(d18:0_24:1)**	650.64	C42 H84 O3 N1	66.6 ± 6.7	51.7 ± 6.0	22.34	1.02E-01
**Cer(d18:0_24:1)**	650.64	C42 H84 O3 N1	4,067.6 ± 229.4	3,145.1 ± 225.3	22.68	**5.14E-03**
**Cer(d18:1_24:0)**	650.64	C42 H84 O3 N1	185,128.9 ± 6,798.8	144,328.2 ± 6,170.5	22.04	**2.56E-05**
**Cer(d18:1_24:0)**	650.64	C42 H84 O3 N1	654.6 ± 112.4	587.5 ± 87.7	10.26	6.39E-01
**Cer(d18:1_24:0)**	650.64	C42 H84 O3 N1	615.2 ± 125.7	600.1 ± 88.5	2.44	9.22E-01
**Cer(d42:1)**	650.64	C42 H84 O3 N1	124.1 ± 68.5	47.9 ± 27.0	61.45	3.67E-01
**Cer(d42:1)**	650.64	C42 H84 O3 N1	14.4 ± 3.5	6.5 ± 0.9	54.68	6.50E-02
**Cer(d18:1_24:0+O)**	666.64	C42 H84 O4 N1	1,767.2 ± 363.4	1,326.4 ± 52.4	24.94	**9.33E-06**
**er(d18:1_24:0+O)**	666.64	C42 H84 O4 N1	2,742.7 ± 213.6	1,830.0 ± 148.8	33.28	**7.56E-04**
**Cer(d18:1_24:1)**	648.63	C42 H82 O3 N1	102.7 ± 9.6	83.3 ± 7.0	18.90	1.07E-01
**Cer(d18:0_24:2)**	648.63	C42 H82 O3 N1	214.5 ± 34.3	183.5 ± 28.3	14.43	4.88E-01
**Cer(d18:1_24:1)**	648.63	C42 H82 O3 N1	54,116.1 ± 2,322.5	46,282.5 ± 2,073.3	14.48	**1.37E-02**
**Cer(d18:2_24:0)**	648.63	C42 H82 O3 N1	31,310.6 ± 1,360.4	26,109.6 ± 1,267.4	16.61	**6.32E-03**
**Cer(d18:1_24:1)**	648.63	C42 H82 O3 N1	786.4 ± 73.1	559.6 ± 49.2	28.83	**1.20E-02**
**Cer(d18:2_24:0+O)**	664.62	C42 H82 O4 N1	1,182.7 ± 97.9	883.0 ± 76.1	25.34	**1.79E-02**
**Cer(d18:2_24:1)**	646.61	C42 H80 O3 N1	10,633.1 ± 487.0	10,017.7 ± 483.4	5.79	3.72E-01
**Cer(d19:0_24:0)**	666.68	C43 H88 O3 N1	948.3 ± 79.2	668.6 ± 64.3	29.49	**7.47E-03**
**Cer(d19:1_24:0)**	664.66	C43 H86 O3 N1	21,388.3 ± 1,251.0	16,325.9 ± 950.1	23.67	**1.82E-03**
**Cer(d18:1_25:0)**	664.66	C43 H86 O3 N1	10,597.7 ± 455.5	8,116.6 ± 344.9	23.41	**4.01E-05**
**Cer(d19:1_24:1)**	662.64	C43 H84 O3 N1	6,852.4 ± 444.2	5,656.9 ± 403.2	17.45	**4.94E-02**
**Cer(d18:2_25:0)**	662.64	C43 H84 O3 N1	2,216.2 ± 108.3	1,717.6 ± 76.2	22.50	**3.20E-04**
**Cer(d18:2_25:1)**	660.63	C43 H82 O3 N1	564.9 ± 48.0	496.5 ± 39.8	12.11	2.76E-01
**Cer(d18:1_26:0)**	678.68	C44 H88 O3 N1	3,944.9 ± 202.7	2,880.0 ± 162.8	27.00	**9.60E-05**
**Cer(d18:1_26:1)**	676.66	C44 H86 O3 N1	1,908.0 ± 79.8	1,501.0 ± 69.9	21.33	**2.34E-04**

Supplementary Table S5. Individual ceramide species in control and PD plasma. LC-MS analysis identified 59 different ceramide species in control (n=44) and Parkinson's disease (n=48) plasma samples. Relative abundance values are given as mean ± standard error of the mean and have been divided by 10^4. Student's t-test was used to compare ceramide levels between the two groups.
